# Frequency of 22q11.2 microdeletion in children with congenital heart defects in western poland

**DOI:** 10.1186/1471-2431-10-88

**Published:** 2010-12-06

**Authors:** Anna Wozniak, Danuta Wolnik-Brzozowska, Marzena Wisniewska, Renata Glazar, Anna Materna-Kiryluk, Tomasz Moszura, Magdalena Badura-Stronka, Joanna Skolozdrzy, Maciej R Krawczynski, Joanna Zeyland, Waldemar Bobkowski, Ryszard Slomski, Anna Latos-Bielenska, Aldona Siwinska

**Affiliations:** 1 Institute of Human Genetics, Polish Academy of Sciences, Strzeszyńska 32, 60-479 Poznan, Poland; 2 Department of Medical Genetics, Poznan University of Medical Sciences, Poland; 3 Department of Paediatric Cardiology and Nephrology, Poznan University of Medical Sciences, Poland; 4 Center for Medical Genetics Genesis, Poznan, Poland; 5 Department of Biochemistry and Biotechnology, Poznan University of Life Sciences, Poland

## Abstract

**Background:**

The 22q11.2 microdeletion syndrome (22q11.2 deletion syndrome -22q11.2DS) refers to congenital abnormalities, including primarily heart defects and facial dysmorphy, thymic hypoplasia, cleft palate and hypocalcaemia. Microdeletion within chromosomal region 22q11.2 constitutes the molecular basis of this syndrome. The 22q11.2 microdeletion syndrome occurs in 1/4000 births. The aim of this study was to determine the frequency of 22q11.2 microdeletion in 87 children suffering from a congenital heart defect (conotruncal or non-conotruncal) coexisting with at least one additional 22q11.2DS feature and to carry out 22q11.2 microdeletion testing of the deleted children's parents. We also attempted to identify the most frequent heart defects in both groups and phenotypic traits of patients with microdeletion to determine selection criteria for at risk patients.

**Methods:**

The analysis of microdeletions was conducted using fluorescence *in situ *hybridization (FISH) on metaphase chromosomes and interphase nuclei isolated from venous peripheral blood cultures. A molecular probe (Tuple) specific to the *HIRA (TUPLE1, DGCR1*) region at 22q11 was used for the hybridisation.

**Results:**

Microdeletions of 22q11.2 region were detected in 13 children with a congenital heart defect (14.94% of the examined group). Microdeletion of 22q11.2 occurred in 20% and 11.54% of the conotruncal and non-conotruncal groups respectively. Tetralogy of Fallot was the most frequent heart defect in the first group of children with 22q11.2 microdeletion, while ventricular septal defect and atrial septal defect/ventricular septal defect were most frequent in the second group. The microdeletion was also detected in one of the parents of the deleted child (6.25%) without congenital heart defect, but with slight dysmorphism. In the remaining children, 22q11.2 microdeletion originated *de novo*.

**Conclusions:**

Patients with 22q11.2DS exhibit wide spectrum of phenotypic characteristics, ranging from discreet to quite strong. The deletion was inherited by one child. Our study suggests that screening for 22q11.2 microdeletion should be performed in children with conotruncal and non-conotruncal heart defects and with at least one typical feature of 22q11.2DS as well as in the deleted children's parents.

## Background

The syndrome of 22q11.2 microdeletions comprises a set of developmental abnormalities, including primarily heart and craniofacial congenital defects. The syndrome is characterised by various phenotypic changes described earlier in DiGeorge, Takao and Shprintzen syndromes. It is the most common microdeletion in the population (1/4000 births) [1]. The 22q11.2DS is inherited in an autosomal dominant manner [2].

In 1993, nosological entities caused by microdeletions in this region were given a common name of CATCH 22, which is an acronym formed by the first letters of the phenotypic traits accompanying this syndrome: C - *cardiac defect*, A - *abnormal face*, T - *thymic hypoplasia*, C - *cleft palate*, H -*hypocalcaemia *[3]. However, the spectrum of clinical features of this syndrome is considerably wider than that indicated by the name CATCH. In the light of the latest investigations, 22q11.2 microdeletion occurs also in patients suffering from the Cayler syndrome and Opitz/GBBB. Therefore, the name CATCH 22 was replaced by 22q11.2 microdeletion syndrome and is known under this name today [4]. Dysmorphy traits are characteristic for the entire 22q11.2 microdeletion syndrome. The most common ones include: elongated face, low-set small dysplastic ears, microstomia, small teeth, congenital tooth or enamel agenesis, almond-shaped eyes, hypertelorism, prominent long bulbous nose, retro- and micrognathia, short neck and characteristic arachnodactyly. The above-listed traits can be clearly pronounced or barely noticeable making phenotypic diagnostics very difficult.

Approximately 180 signs of the 22q11.2DS have been described to date. These include abnormalities of the urinary-reproductive, skeletal, gastrointestinal, nervous and immunological systems as well as psychiatric disturbances. Moreover, considerable changes in the phenotype expression are observed in persons with a deletion of the same size, belonging to the same family or even in monozygotic twins [5]. In the light of these facts, it is difficult to determine the correlation between genotype and phenotype in patients with 22q11.2DS [6]. Causes can be attributed to: heterogeneity, pleiotropy, variations in mutation penetration, varying impact of environmental effects as well as other factors or events affecting the foetus [7].

In 90% of patients, the identified microdeletion covers the region of 3 Mbp, encoding 30 genes, referred to as the typically deleted region (TDR), e.g. *DGCR6, PRODH, IDD, TUPLE1, UFDL1, TBX1, ZNF74, CRKL*, whereas microdeletion of 1.5 Mbp including 24 genes has been found in 8% of patients. A minimal DiGeorge critical region (MDGCR) covers about 0.5 Mbp and several genes: *CTP, CLTD, TUPLE1, UFD1, TBX1, CDC45 *[8]. The remaining 2% include patients with other chromosomal aberrations (e.g. del10p) [9]. Interestingly, the intensity of clinical symptoms is not correlated with the size of microdeletions [10]. Atypical deletions in the 22q11.2 region are rare. In patients with chromosome 22q11.2 microduplication syndrome, similar phenotype can be found.

The 22q11.2 microdeletion accounts for 5% of all heart defects in newborns [11]. In approximately 10-20% of patients the 22q11.2 microdeletion does not originate *de novo *but is also found in one of the parents who was not earlier identified as the mutation carrier [12]. A phenotype similar to the 22q11.2DS may also result from environmental factors such as diabetes or alcoholic disease affecting the foetus as well as from increased or decreased levels of retinoic acid (vitamin A) in the mother's organism [13].

The aim of this study was to determine the frequency of the 22q11.2 microdeletion in (i) 87 children with a congenital heart defect (37 conotruncal and 50 non-conotruncal cases) and at least one additional 22q11.2DS feature, (ii) a group of the deleted children's parents. We also attempted to establish the correlation of the most frequent heart defects in both groups and characteristic phenotype in patients with 22q11.2 microdeletion to determine selection criteria for at risk patients.

## Methods

FISH (fluorescence *in situ *hybridization) method was used in a group of 87 children with a congenital heart defect at least one additional 22q11.2DS feature: facial dysmorphy, thymic or parathyroid gland hypoplasia or aplasia, cleft palate or lip and hypocalcaemia during the neonatal period or accompanying abnormalities of urinary-reproductive, skeletal, gastrointestinal or nervous systems. The patients were divided into two groups: with conotruncal (n = 35) and non-conotruncal heart defects (n = 52). The examined group comprised 39 girls and 48 boys. The average age of all examined children was 2 years (0-6 years); thirty one patients (35.63%) were newborns.

Additionally, seventeen of the deleted children's parents were included in the study. In one case, only one of the parents was examined. Having obtained a written consent of the parents, blood samples for genetic analyses were taken from the patients. The following analyses were performed: case history analysis, cardiologic examination and detailed pedigree and karyotype analysis as well as object examination together with phenotype assessment.

In all cases, the heart anatomy and physiology were diagnosed by echocardiography. In complex cardiac defects cardiac catheterization was performed. The thymus and parathyroid glands were visualized by ultrasound, and CT or MRI. Assessment of parathyroid dysfunction included detailed clinical history regarding the symptoms of hypocalcaemia and the following biological features: concentration of total calcium (normal range 2.2-2.6 mmol/l), phosphate (normal range 0.8-1.45 mmol/l) and ionized calcium (normal range 1.17-1.30 mmol/l). Low calcium levels in the blood indicated hypocalcaemia (total calcium <2.2 mmol/l; ionized calcium <1.17 mmol/l).

Heparinised lymphocytes of venous peripheral blood (0.5-1 ml each) stimulated with phytohemagglutinin were used for the cytogenetic studies. Isolated cells were grown and harvested in accordance with standard procedures. The obtained chromosome preparations were stained with the GTG standard method [14]. Fluorescence *in situ *hybridization using DiGeorge (Tuple) Region Probe Dual Colour (Qbiogene) was performed according to the procedure established by the manufacturer. This probe is specific to the *HIRA (TUPLE1, DGCR1*) region at 22q11. The hybridization signals were documented with a Zeiss-Axiophot fluorescent microscope and analysed with CytoVision 3.52 software.

The study was approved by the Ethic Committee at the Poznan University of Medical Sciences.

## Results

Microdeletions in the region q11.2 of the 22 chromosome were identified in the group of fourteen patients - thirteen children with a congenital heart defect (conotruncal and non-conotruncal) and at least one additional trait from the 22q11.2DS spectrum (14.94% of children from the analysed group) and one parent of a deleted child. Patients were classified into 2 groups based on the conotruncal and non-conotruncal cardiac defects. Microdeletion of 22q11.2 occurred in 20% and 11.54% of the first (7/35 conotruncal patients) and the second group of children (6/52 non-conotruncal patients) respectively. The congenital heart defects and details of phenotypic abnormalities are described in Table 1.

**Table 1:** Characteristics of the children with microdeletion 22q11.2

Patients	Cardiac defect	Extra cardiac defects
1	ToF+ASDII+MAPCAS	Micrognathia, palatopharyngeal insufficiency, thymic and parathyroid gland hypoplasia, hypocalcaemia, choanal atresia, club foot, ectopic kidney
2	ASDII+VSD	Thymic gland hypoplasia
3	VSD	Thymic gland hypoplasia
4	ToF	Blepharophimosis, prominent and bulbous nose, narrow lip red, dysplastic ears, arachnodactylia, foot deformation
5	ASDII	Blepharophimosis, antimongoloid arrangement of lid slits, narrow lip red, narrow lips, micrognathia, low-set ears, dysplastic ears, palatopharyngeal insufficiency, arachnodactylia, mild mental retardation, ostium of the ureter atresia, cryptorchism
6	ToF	Hypertelorism, blepharophimosis, prominent and bulbous nose, narrow lip red, micrognathia, dysplastic ears, club foot
7	VSD+PDA+FO	Micrognathia, retrognathia, dysplastic ears, hypocalcaemia, arachnodactylia, increased muscular tension, incorrect distribution of palm fingers
8	ToF	Narrow lip red, low set ears, arachnodactylia
9	ToF	Dysplastic ears, arachnodactylia
10	ToF	Prominent and bulbous nose, micrognathia, low set ears, dysplastic ears
11	ASDII+VSD	Blepharophimosis, antimongoloid arrangement of lid slits, broad nose, micrognathia, retrognathia, dysplastic ears, arachnodactylia, delayed psychomotor development
12	ToF	Antimongoloid arrangement of lid slits, microphthalmia, prominent and bulbous nose, dysplastic ears, thymic gland hypoplasia, arachnodactylia
13	VSD	Micrognathia, dysplastic ears, arachnodactylia

ASDII - atrial septal defect II; FO - foramen ovale; MAPCAS - major aortopulmonary collaterals; PDA - patent ductus arteriosus; ToF - tetralogy of Fallot; VSD - ventricular septal defect;

The analysed group of patients with the microdeletion comprised nine girls and four boys. The mean age on the day of examination was one year and two months, with seven patients diagnosed in the first month, three - in the first year and the remaining patients in the second, third and sixth year of life. The performed karyotype analysis of all patients did not reveal any abnormalities regarding the number and structure of chromosomes.

Microdeletion of 22q11.2 was detected in 1/17 parents of nine children. The parent with microdeletion did not have any diagnosed heart defects and exhibited only slight dysmorphism- ear dysplasia and arachnodactyly. The above-mentioned features were more pronounced in his child. Microdeletion was not detected in any other parent, which indicates that in the case of 7 children 22q11.2 microdeletion originated *de novo*.

Pedigree analysis of all the patients revealed the family history with a congenital heart defect in 14 cases (16.09%). Parents of the deleted children did not harbour any congenital defects including heart or other principal trait characteristics for the 22q11.2DS.

## Discussion

Microdeletion of 22q11.2 was detected in 14.94% of the examined patients (Table 2). Other authors indicate wide range of prevalence rate depending on different selection criteria [15-19]. The frequency of 20% and 11.54% was observed in the first and second group of patients respectively. The published data reveal frequency of 22q11.2 microdeletion in conotruncal and dysmorphic patients at the level of 15.8% and 25% respectively [19,20]. Our data shows increased prevalence of 22q11.2 microdeletion (11.54%) in patients with non-conotruncal heart defect and at least one typical feature of this syndrome compared to other research studies whose results equalled 8.3% and 10% [18,21]. As postulated by Tobias et al., these results probably contribute to the expansion of the clinical and phenotypic criteria used in screening of patients at risk for 22q11 microdeletion [22]. It should be mentioned that data which indicate occurrence of this deletion in patients with conotruncal or non-conotruncal heart defects without characteristic phenotype show very low percentage of the mutation (0%, 5.7%, 6.5%) [18,23,24]. However, the dysmorphic features may be overlooked and extracardiac defects (i.e. mental retardation) may appear later.

**Table 2:** Prevalence of phenotypic characteristics in patients with microdeletion 22q11.2 in comparison to the published data

Feature	Results of our study	Other authors
The prevalence of 22q11.2 microdeletion	14.96%	48% (Iserin i wsp., 1998)
		29% (Goldmuntz i wsp., 1993),
		11.62% (Yakut i wsp., 2006),
		9.4% Barisic et al.2008
		6.16% Halder et al., 2010
		
Presence of heart defect	100%	75% (Ryan et al., 1997)
		
The most frequent conotruncal heart defect	ToF 53.84%	ToF 100% Halder et al., 2010
		ToF 66.66% Barisic et al.2008
		ToF 17% Ryan et al., 1997
		
The most frequent non- conotruncal heart defect	ASDII+VSD 15.38%	16.66% Barisic et al, 2008
	VSD 15.38%	14% Ryan et al., 1997
		
Face dysmorphia	84.51%	50-100% Yakut et al, 2006
		
Cleft palate	0%	9% Rayan et al, 1997
		0% Yong et al, 1999
		
Palatopharyngeal insufficiency	15.38%	32% Persson et al, 2003
		32% Ryan et al., 1997
		
Thymic hypoplasia	30.76%	91% Oskarsdottir et al, 2005
		
Hypocalcaemia	15.38%	32.89% Choi et al, 2005
		60% Ryan et al., 1997
		
Mental retardation	1%	35-40% Ryan et al, 1997
		
Non-typical feature	61%	50% Oskarsdottir et al, 2005

The obtained frequencies of occurrence of the remaining heart defects were similar to the data derived from the European multi-centre research on the spectrum of phenotypic traits in patients with 22q11.2DS as well as from published literature data. As indicated in the literature the most frequent heart defects in children with 22q11.2DS include: tetralogy of Fallot, ventricular septal defect, interrupted aortic arch, pulmonary valve atresia with ventricular septal defect, persistent truncus arteriosus, transposition of the great arteries and atrial septal defect [17,18,25].

Facial dysmorphia is another typical feature for 22q11.2DS is facial dysmorphia. Our study indicated dysmorphic nature of discreet to strong expression, which is identical to the results of other researchers (Table 1, 2) [25,17,26]. In newborns with microdeletions, problems with milk suckling and regurgitation, associated with palatopharyngeal insufficiency, are often observed. However, the problems with food intake are not necessarily caused by cleft lips and palate. It has been estimated that delayed speech development and rhinolalia may occur in up to 50% of patients in this group [27].

In many children with 22q11.2DS and disturbed thymus function, decreased immunity, increased proportion of complications after heart operations and higher mortality rate are observed [28]. For this reason it is believed that these children should be vaccinated at a later date than recommended in the vaccination schedule. Only in about 5% of patients with the 22q11.2DS microdeletion bone marrow transplantation or thymus transplantation might be necessary [29]. It has also been shown that thymus dysfunction predisposes patients with the deletion to different autoimmune diseases, i.e. juvenile arthritis, thrombocytopenia and Graves-Basedov disease [30]. In our group of patients, thymic hypoplasia coexisted with typical dysmorphic changes only in one child. In two patients with thymic hypoplasia, no signs of facial dysmorphy were observed. In two remaining cases the changes were considerably milder.

Another sign complicating the course of the neonatal period in the examined patients with 22q11.2DS was hypocalcaemia [31]. It should be emphasised that in our studies, thymic or parathyroid gland hypoplasia as well as hypocalcaemia occurred exclusively in the group of children with the 22q11.2 microdeletion. A similar relationship was found in experiments conducted by Koch et al., who carried out comparative investigations of calcium and thyroid hormone levels in two groups of adults with and without microdeletion. One group comprised patients with confirmed 22q11.2DS and congenital heart defect secondary to the conotruncal heart defect (tricuspid atresia, tetralogy of Fallot, pulmonary atresia with ventricular septal defect, interrupted aortic arch type B), while the other group was formed by patients with similar heart defects but without confirmed microdeletions [31,32]. Therefore, thymic or parathyroid gland hypoplasia coexisting with a congenital heart defect should indicate the need to carry out genetic examinations for 22q11.2DS.

Currently, more attention is being devoted to problems of psychiatric, psychological and behavioural nature in patients with the 22q11.2 microdeletion. In the examined group of children with 22q11.2DS, one patient was diagnosed with slight mental retardation and another with delayed psychomotor development. However, it is worth emphasising that the majority of children were examined during the neonatal period when it is difficult to determine unequivocally if the psycho-physical development of a child is normal. According to investigations carried out by other researchers, the average value of the intelligence quotient (IQ) in deleted patients amounts to 75 and abnormal development is observed in approximately 35-40% of patients with microdeletion [33]. Most frequently, problems in learning and abstract thinking are observed [34]. Therefore, in properly developed newborns and infants with 22q11.2DS, it is impossible to rule out their incorrect development in the future, which could be attributed to the 22q11.2 microdeletion. This remark also refers to psychiatric diseases. Increased incidence (up to 20%) of depressions, schizophrenia and bipolar disorder was demonstrated in adult patients with 22q11.2DS. Hemizygotic deletion on chromosome 22 was found in 2-5% of patients suffering from schizophrenia [35].

Diagnosis based on a clinical picture exclusively may be difficult due to the fact that 22q11.2 microdeletion phenotype is not always fully developed, dysmorphy may vary from very pronounced to quite discreet and patients may show non-typical signs. Our studies indicate that it is necessary to perform the analysis of the 22q11.2 microdeletion not only in children with congenital heart defect secondary to conotruncal heart defect but also in patients with other heart defects coexisting with at least one feature from the 22q11.2DS spectrum.

Literature data reveal that about 10-20% of patients inherit 22q11.2 microdeletion from one of the parents [12]. Frequently, the phenotype of affected parents exhibits none or only discreet traits of 22q11.2DS. This can probably be attributed to variable levels of penetration of this mutation. It is evident from experiments carried out on monozygotic twins that the 22q11.2 microdeletion of identical size can cause varying phenotype consequences [36]. On the other hand, studies conducted by Adeyinka et al. suggested familial origin of small-sized deletions. Exceptionally large differences may relate to congenital heart defects [37]. Genetic analyses of the 22q11.2 region carried out in two groups of patients with familial incidence of congenital heart defects revealed that this change is inherited in 44% and 13% of patients respectively [27,38].

The high frequency of 22q11.2 microdeletions originating *de novo *might suggest considerable genomic instability in this region of chromosome 22. The genetic mechanism behind the microdeletion is based on nonallelic homologous recombination (NAHR). The same aetiology has been observed in the case of other microdeletions - Angelman (15q11-q13 microdeletion), Prader-Willi (15q11-q13 microdeletion) and Williams-Beuren (7q11.2 microdeletion) syndromes [7].

It is believed that some of the patients with the 22q11.2DS phenotype do not have a chromosomal microdeletion but that they are likely to be carriers of mutations in one or more genes [8]. This fact has been observed in other microdeletion syndromes as, for example, William's and Wolf-Hirschhorn's syndromes (4p16.3 microdeletion). In the majority of cases, the 22q11.2 microdeletion *locus *comprises approximately 30 genes. Comprehensive functional studies on animal models revealed that *TBX1 *is the only gene with haploinsufficiency that results in the occurrence of a phenotype characteristic for the 22q11.2DS [39]. *TBX1 *gene encodes phylogenetically conservative transcription factor which takes part in the regulation of developmental processes. The extensive mechanisms in which it is involved create many possible ways for the development of abnormalities caused by haploinsufficiency - lack, excess or incorrect structure of the *TBX1 *gene. The discovery of the mutation in the *TBX1 *gene in a patient with the microdeletion phenotype and the recognition of its impact on the TBX1 protein accelerated the understanding of the molecular basis of 22q11.2 microdeletion syndrome [11].

It is possible to diagnose the 22q11.2 microdeletion using other diagnostic methods than FISH, ie. *high resolution comparative genomic hybridization *(HR-CGH), *multiplex ligation-dependent probe amplification *(MLPA), *short tandem repeats *(STR) as well as *quantitative polymerase chain reaction *(qPCR). These methods enable researchers to estimate the size of microdeletions, determine chromosomal breakpoints and perform segregation analysis [40].

Regardless of the methods applied to diagnose 22q11.2DS, early diagnosis is crucial. This is why the possibility of prenatal identification of congenital heart defects begins to play an important role. The result of echocardiographic examinations confirming a heart defect in the foetus can frequently give the baby a chance to survive as adequate care can be provided. In addition, the result could qualify newborns to a risk group and indicate the need to perform genetic analysis. This assumption was confirmed by investigations carried out by Moore et al. who prenatally diagnosed foetuses by means of USG. They demonstrated that 41% of foetuses with congenital heart defect had an incorrect karyotype and 3% had 22q11.2 microdeletion [41].

Long-term studies carried out in several research centres indicate that children with congenital heart defects are most likely to undergo examinations for microdeletions on chromosome 22 while being the patients on paediatric cardiology wards, even though they are under the care of other specialists [28]. Our studies also confirmed the above data as 70% of patients that we diagnosed with the 22q11.2 microdeletion were referred to the examinations by the physicians from the Department of Paediatric Cardiology and Nephrology of the Poznan University of Medical Sciences.

## Conclusions

There is a wide spectrum of phenotypic characteristics that occur in patients with the 22q11.2DS. This syndrome is characterized by a highly variable expression of phenotype, ranging from discreet to quite strong. ToF was the most frequent conotruncal heart defect in the group of children with 22q11.2 microdeletion, while ASDII+VSD and VSD were the most common in the non-conotruncal group. Our studies indicate that patients with moderate heart defects (atrial or ventricular) or frequently observed complete absence of dysmorphism exhibit a typical but rare thymic hypoplasia. In one familial case of the 22q11.2DS, no cardio-vascular defect was identified in the parent who was the microdeletion carrier, despite the fact that tetralogy of Fallot was diagnosed in the child with the same mutation.

Many patients with the 22q11.2DS additionally suffer from accompanying defects, primarily, associated with urinary-reproductive, skeletal, gastrointestinal, nervous systems and the organ of sight. Therefore, at various stages of life they will require multi- and highly-specialized care of cardiologists, cardiosurgeons, immunologists, orthopaedists, psychologists and psychiatrists. Such long-term and specialized care over patients with the 22q11.2DS is provided, for example, in Goteborg, Tokyo or Philadelphia [7]. It should also be emphasised that patients with this syndrome as well as their families should be under genetic supervision. Our observations suggest that the criteria in searching for microdeletion 22q11.2 should be expanded and applied in patients with conotruncal and non-conotruncal congenital heart defects and at least one typical feature of this syndrome (facial dysmorphy, thymus hypoplasia, cleft palate or hypocalcaemia) as well as in the deleted children's parents.

## Competing interests

The authors declare that they have no competing interests.

## Authors' contributions

Being the principal investigator of the project, AW planned and organized the study, conducted FISH analysis and prepared the manuscript, DW-B, MW, RG, AM-K, TM, MB-S, MK enlisted the patients and provided clinical data. JS, JZ performed FISH. AL-B, AS, WB, RS verified and interpreted clinical and genetic results.

## Pre-publication history

The pre-publication history for this paper can be accessed here:

http://www.biomedcentral.com/1471-2431/10/88/prepub
